# Coacervation in polyzwitterion-polyelectrolyte systems and their potential applications for gastrointestinal drug delivery platforms

**DOI:** 10.1038/s41467-022-29851-y

**Published:** 2022-04-26

**Authors:** Khatcher O. Margossian, Marcel U. Brown, Todd Emrick, Murugappan Muthukumar

**Affiliations:** 1 Department of Polymer Science and Engineering, University of Massachusetts, Amherst, MA 01003 USA; 2 Rush Medical College, Rush University Medical Center, Chicago, IL 60612 USA

**Keywords:** Polymers, Self-assembly, Polymers, Molecular medicine

## Abstract

Traditionally, complex coacervation is regarded as a process whereby two oppositely charged polyelectrolytes self-assemble into spherical droplets. Here, we introduce the polyzwitterionic complex, “pZC”, formed by the liquid-liquid phase separation of a polyzwitterion and a polyelectrolyte, and elucidate a mechanism by which such complexes can assemble using theory and experimental evidence. This system exhibits orthogonal phase behavior-it remains intact in acidic conditions, but disassembles as the pH increases, a process governed by the acid-base equilibria of the constituent chains. We relate the observed phase behavior to physiological conditions within the gastrointestinal tract with a simulation of the gastroduodenal junction, and demonstrate using video microscopy the viability of polyzwitterionic coacervates as technologies for the pH-triggered release of cargo. Such a system is envisaged to tackle imminent problems of drug transport via the oral route and serve as a packaging solution to increase uptake efficiency.

## Abstract (web summary)

Coacervation is widely studied as potential drug delivery platform, but formation of coacervates at lower pH ranges, such as found in the gastrointestinal tract, remains challenging. Here, using theory and experimental methods, the authors demonstrate the formation of a polyzwitterionic complex, formed by coacervation of a polyzwitterion and a polyelectrolyte, exhibiting orthogonal phase behavior under physiological low pH conditions.

## Introduction

Complex coacervates have been measured and studied extensively since they were first described in the early 20th century^1–7^. Typically, they are formed by the association of two oppositely charged polyelectrolytes, and their self-assembly is driven by the entropically favorable release of counterions and solvent into the bulk mixture^8–11^. This process results in liquid-liquid phase separation between a polymer-rich dense phase, and a polymer-poor dilute supernatant. When viewed using optical microscopy, the hallmark of coacervation is the presence of smooth, mobile, and coalescing spherical droplets. Although many variations on this theme exist in the literature—biologically-inspired monomers that form specific three-dimensional structures, incorporation of charged groups into block copolymers, sequential and chiral patterning, and many other physicochemical alterations—the majority of the surveyed work relies on the association of two macromolecular chains that are of uniformly negative or positive charge^12–20^.

**Fig. 1:**
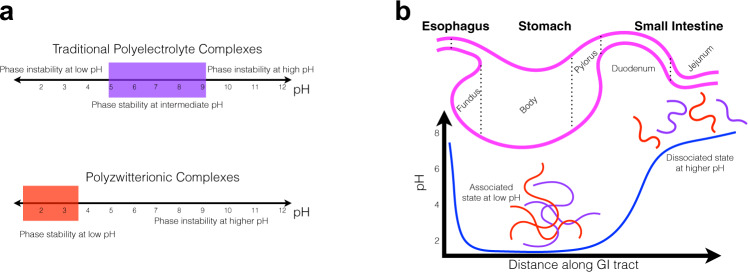
Orthogonal phase behavior of polyzwitterionic complexes and their applicability to gastrointestinal cargo delivery. **a** Comparing the phase behavior of traditional polyelectrolyte complexes (top, purple box) with polyzwitterionic complexes (bottom, red box). In traditional systems, phase stability (i.e., the parameter space in which stable complexation can occur) is present at intermediate pH values (purple box) because of the p*K*_*a*_ values of the constituent chains. Maximal complexation occurs when both positive and negative chains are maximally charged, typical values for which are found within the purple box. In contrast, with polyzwitterionic complexes, stable spherical droplets are formed when the pH is in a more acidic range (red box). **b** Schematic diagram of pH variation along the GI tract and design principle. Inside the acidic environment of the stomach, polyzwitterionic complexes would remain in their associated state. As they move into the duodenal space, the pH (blue line) rapidly increases to a value of 6–8, which would result in de-complexation.

Classically, coacervate solutions are most stable within a pH range that allows both cationic and anionic species to remain in a maximally charged state^9,21–23^. This phenomenon is illustrated by the purple box in Fig. 1a, which denotes the range in which coacervates are stable in traditional polyelectrolyte systems. Outside this range, there is insufficient ionization of one chain to promote coacervation. However, it is desirable to push this box to different ranges, for example in order to control the retention and release characteristics using pH as a chemical trigger. Moving the box leftwards (Fig. 1a, red box) to lower pH ranges presents opportunities in the physiological arena.

An important example in which such a scenario is beneficial is in the gastrointestinal (GI) tract. Considering the large pH gradient between the stomach and the intestines (Fig. 1b), platform technologies that control the retention and release of material using this large gradient can improve the delivery of cargo to areas distal to the stomach^24^. This problem is of great medical interest because currently, many oral drug delivery technologies suffer from significant degradation upon exposure to the harsh gastric environment. As a result, many modern therapeutics cannot be formulated as orally-delivered drugs and can instead only be delivered intravenously^24–26^. Avoiding gastric degradation is especially tantalizing because the small intestine is the site of greatest and most efficient absorption in the GI tract^27–29,30,31^. Furthermore, intestinal conditions are much gentler, since the production of basic secretory fluids neutralizes its luminal pH^32–35^. Hence, there is an opportunity for drug delivery technologies to take advantage of this significant pH differential by using it as a chemical trigger to release encapsulated cargo.

Our idea is based on the theory of complexation between charged macromolecules. The two major contributions to inter-molecular complexation among polar and charged macromolecules are electrostatic interactions among all polar and charged repeat units of the complexing chains and entropy gain due to the release of counterions during complexation. Let us consider complexation between a host macromolecule and a guest macromolecule. If both host and guest are uniformly charged as in the case of complexation between polycations and polyanions, the electrostatic interaction is the strongly attractive charge-charge interaction, with counterion release further augmenting the process. On the other hand, if the host is made of essentially polar repeat units with only very few charges, then it can be treated as a chain of dipoles. If the guest is uniformly charged, the electrostatic interaction between the dipolar host and charged guest is a dipole-charge interaction. Complexation in this situation too is facilitated by the dominant driving force from counterion release. If both the host and guest are dipolar chains, then the electrostatic interaction energy is a dipole-dipole interaction, which is much weaker than the dipole-charge interaction. Furthermore, there is no additional driving force from counterion release if both the host and guest are dipolar. Therefore, we expect the spontaneously formed coacervate complex from a dipolar chain and a charged chain to dissolve when the condition is such that the dipole-charge interaction is switched to a dipole-dipole interaction.

To be specific, let the host molecule be poly(acrylic acid) (p*K*_*a*_ ≃ 6.2) and the guest be the polyzwitterion poly(2-methacryloyloxyethyl phosphorylcholine) (pMPC) (p*K*_*a*_ ≃ 2.3 for the phosphoryl group). Under the experimental conditions of interest here, namely pH < 4, polyacrylic acid can be treated as a dipolar chain. For pH < 4, the polyzwitterion is essentially a polycation, and for pH > 4, it is a dipolar chain. Since the dipole-charge interaction, accompanied by counterion release, results in complexation and mere dipole-dipole interactions are not strong enough to lead to complexation, we envisage a mechanism of devising a change from a complexed state to an uncomplexed state in the above mentioned pH range, by making the zwitterionic repeat unit of the guest charged at lower pH and dipolar at higher pH.

**Fig. 2:**
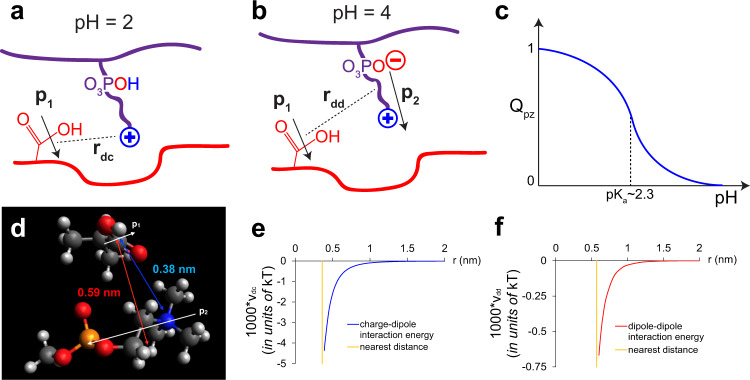
Schematics of the electrostatic interaction between pAA and pMPC. **a** At low pH (~2), the repeat unit of pAA (red) is deionized and acts as a dipole, while the phosphoryl group of the zwitterionic repeat unit of pMPC (purple) is deionized so that the monomer acts as a positive charge, resulting in dipole-charge interaction at separation distance *r*_*dc*_. This attractive energy, in combination with counterion release, leads to complexation. **b** Schematic of the electrostatic interaction between pAA and pMPC at pH ~ 4. The pAA monomer is again deionized and acts as a dipole. However, the phosphoryl group of pMPC is ionized so that the monomer is electrically neutral and thus, also acts as a dipole. The net effect is a weak dipole-dipole interaction between pAA and pMPC. **c** Since the *pK*_*a*_ of the phosphoryl group is 2.3 and the charge on the ammonium group is fixed, the net charge *Q*_*pZ*_ of the zwitterion changes from +1 to zero as the pH is increased from 2 to 4. **d** Snapshot of the packing model of a repeat unit of pAA and that of pMPC from the Avogadro software. The closest distance between the dipolar pAA and the charged pMPC is 0.38 nm; for the dipolar zwitterion it is 0.59 nm. **e** Plot of the dipole-charge interaction energy per contact (blue curve) as a function of their separation distance (*ℓ*_*B*_ = 0.7 nm, *p*_1_ = 0.0354, and *κ* = 0.329 nm^−1^ at pH 2). The orange vertical line represents the nearest distance between the charge and the dipole, 0.38 nm. **f** Plot of dipole-dipole interaction energy per contact (red curve) as a function of their separation distance; *ℓ*_*B*_ = 0.7 nm, *p*_1_ = 0.0354, *p*_2_ = 0.39, *κ* = 0.0329 nm^−1^ at pH 4. The orange line represents the nearest distance between dipoles, 0.59 nm.

Quantitative computation of the energetics associated with coacervate complexation involving polyzwitterions is difficult due to rich chemical details and the present lack of knowledge about the local dielectric constant that mediates the various electrostatic interactions in the system. However, a qualitative and instructive account of the energetics for the above specific system can be formulated as follows. Focusing on the neighborhood of a pair of complementary repeat units, the situations corresponding to low pH and high pH are cartooned in Fig. 2a, b, respectively. At both low pH and high pH considered here, the acrylic acid repeat unit is treated as a dipole with dipole moment ${\overrightarrow{{{{{{\bf{p}}}}}}}}_{1}$. However, at low pH, the zwitterionic repeat unit carries a net positive charge due to protonation of the phosphoryl group; at higher pH, the zwitterionic repeat unit is a dipole with dipole moment ${\overrightarrow{{{{{{\bf{p}}}}}}}}_{2}$. The net charge of the MPC group (Q_*pz*_) is sketched in Fig. 2c as a function of pH. Therefore, the electrostatic energy of complexation at low pH is the dipole-charge interaction energy (*v*_*dc*_) and that at higher pH is the dipole-dipole interaction energy (*v*_*dd*_). Averaging over all allowed orientations of the dipoles separated by distance *r*, *v*_*dc*_(*r*) and *v*_*dd*_(*r*) are given by

1 $$\frac{{v}_{dc}(r)}{{k}_{B}T}=-\frac{1}{6}\frac{{\ell }_{B}^{2}{p}_{1}^{2}}{{r}^{4}}{e}^{-2\kappa r}{\left(1+\kappa r\right)}^{2},$$

and

2 $$\frac{{v}_{dd}(r)}{{k}_{B}T}=-\frac{1}{3}\frac{{\ell }_{B}^{2}{p}_{1}^{2}{p}_{2}^{2}}{{r}^{6}}{e}^{-2\kappa r}\left[1+2\kappa r+\frac{5}{3}{(\kappa r)}^{2}+\frac{2}{3}{(\kappa r)}^{3}+\frac{1}{6}{(\kappa r)}^{4}\right],$$

where *k*_*B*_*T* is the Boltzmann constant times the absolute temperature, *ℓ*_*B*_ is the Bjerrum length ( = *e*^2^/(4*πϵ*_0_*ϵk*_*B*_*T*), *e* is the electronic charge, *ϵ*_0_ is the permittivity of vacuum, *ϵ* is the effective dielectric constant of the medium), and *κ* is the inverse Debye length^43^.

A model of the nearest distances between the acrylic acid dipole and charged ammonium group and that between the acrylic acid group and the dipolar zwitterion is given in Fig. 2d, based on the Avogadro software. Interaction energies were minimized from numerous starting configurations using the MMFF-94 (Merck Molecular Force Field) algorithm, and the calculated geometrical configurations are expected to approximate global minima. As an estimate, these distances are taken to be 0.38 nm and 0.59 nm, respectively. Taking *ℓ*_*B*_ = 0.75 nm (corresponding to aqueous solutions at room temperature), *κ* = 0.329 nm^−1^ at pH 2 and *κ* = 0.0329 nm^−1^ at pH 4, *p*_1_/*e* = 0.0354 nm (corresponding to the dipole moment of acrylic acid as 1.7 D), and estimating the dipole moment of dipolar zwitterion as 0.39 e (corresponding to 18.74 D), typical *r*-dependence of *v*_*dc*_ and *v*_*dd*_ calculated from the above equations are shown in Fig. 2e, f. Here, the cutoff distances for *v*_*dc*_ and *v*_*dd*_ are taken from Fig. 2d as 0.38 nm and 0.59 nm, respectively.

As seen from Fig. 2e, f, the pairwise interaction energy of a charge and a dipole is an order of magnitude stronger than that for a pair of dipoles. The latter is too weak to enable any complexation at room temperature. For the former process of complexation involving a charged group, there is an additional significant driving force from the release of counterions from the associating monomers. Therefore, we expect complexation at lower pH < 4 and no complexation at higher pH > 4. Even though the above theoretical argument is only conceptual, without very detailed accounting of subtleties such as water reorganization and local dielectric constant, it provides a sound guideline for the observed phenomena outlined below. It is to be noted that the amine group of MPC could displace the proton of the acrylic acid monomer during complexation, thus making an ion pair in the complexed state. In this situation, the complex is made more stable due to stronger charge-charge (compared to dipole-charge) interactions and the concurrent gain in translational entropy of the released proton. That being said, the key variables are the pH-tunable charge/dipole moment of the repeat units of the host and guest, ionic strength, and polarizability of the medium. With the above concepts in mind, it is possible to design functional polyzwitterion systems by selecting for certain properties in these materials.

In order to exhibit the feasibility of our idea, and at the same time adapt it to a relevant problem, let us return to the need to protect cargo for delivery into the gastrointestinal tract. Some relevant parameters include the pH of the stomach, which is close to 2, and the pH of the duodenum, which increases to a value of 5–8^36,37^. Additionally, the temperature of the GI tract typically varies between 35–40 ^∘^C^37,38^. With these physiological parameters in mind, various functional chemistries can be anticipated to be useful in this situation.

Using the above guidance from nature, we can justify our earlier choice of pMPC and pAA to explore the feasibility of this new class of materials, comprised of interacting chains of zwitterionic and anionic polymers, with respect to their specific applicability to the retention and release of cargo inside the gastrointestinal tract. Furthermore, our anionic polymer of choice, pAA, has a simple structure, wide availability, and is also a common food additive recognized by the United States Food and Drug Administration. We term these materials “pZCs”, short for “polyzwitterionic complexes”. In order to survive the harsh conditions inside the stomach, our expectation is that complexation occurs at low pH values, and dissociation at higher pH values.

In addition to this pH-dependent behavior, complexes must remain intact across a wide temperature range, encompassing ambient conditions in both a typical lab or household environment, as well as in physiologically relevant conditions exceeding 35 ^∘^C. These compounded challenges serve as an obstacle in orally-delivered drug design, since the exposure of most drugs to the stomach contents causes rapid disintegration, reducing their uptake efficiency. Hence, blood concentrations of an ingested drug are always lower than the initial dosage would suggest. Simply raising the dosage is an unacceptable solution for very expensive drugs, or for drugs that cause acute toxicities when present at sufficiently high local concentrations^39^. For these reasons, drug packaging designed expressly for the small intestine (particularly the duodenum) has remained a frontier in pharmaceutical research. If cargo did arrive into the small intestine unmolested by the gastric environment, the available blood concentration for that cargo would be much higher than under typical circumstances. Furthermore, if it becomes possible to shield sensitive cargo from corrosion by the stomach, new opportunities will emerge within the context of packaging advanced therapeutics^39–42^.

In this work, we demonstrate the formulation of a polymeric system that undergoes liquid-liquid phase separation to self-assemble into droplets. These pZC droplets represent a new class of materials distinct from traditional coacervates, since their composition departs from that of traditional, oppositely charged, polyelectrolytes. We show phase behavior in low pH conditions that would destroy most electrostatic assemblies, exhibit nonlinear stoichiometry- and pH-dependent behavior, and reveal the spherical morphology of pZC droplets. Next, we discuss a mechanism that explains this phenomenology and links these observations to applications in gastrointestinal physiology. Finally, we describe a method by which the non-equilibrium, responsive properties of these materials can be harnessed and viewed in real time using video microscopy, within conditions that mimic relevant aspects of the duodenal environment. Our pZCs satisfy the key requirements of gastrointestinal drug delivery outlined above, and provide a blueprint by which strategies for drug delivery into the GI tract can be facilitated.

## Results

### Liquid-liquid phase separation in polyzwitterion-polyelectrolyte solutions

**Fig. 3:**
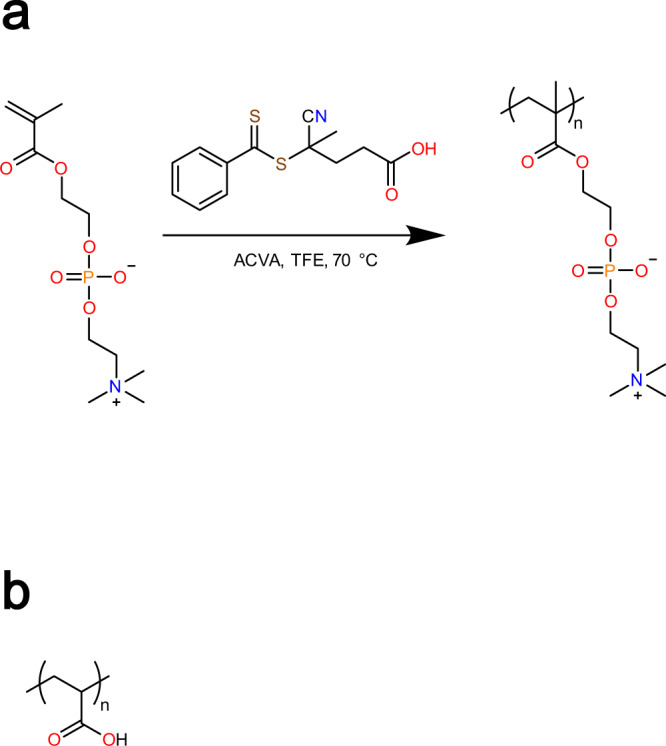
Materials used in the study. 22 kDa pMPC (**a**) was synthesized from monomeric MPC, and mixed with pAA (**b**) of 50 kDa molecular weight to form complexes.

To assess the phase behavior of poly(2-methacryloyloxyethyl phosphorylcholine)-poly(acrylic acid) (structures depicted in Fig. 3, henceforth abbreviated as “pMPC-pAA”) droplets, we tested the effect of the mixing ratio of the two components on the turbidity of the resulting solution.

**Fig. 4:**
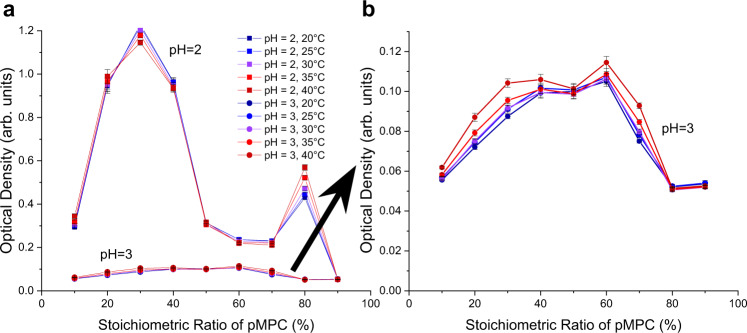
Stoichiometry of pMPC:pAA vs. turbidity at pH 2 (squares) and 3 (circles), plotted over a temperature range from 20–40 ^∘^C (blue to red, respectively). **a** Data from the pH 2 samples demonstrate a peak value at a 30:70 mixing ratio. By comparison, the pH 3 data at the bottom of graph (**a**) exhibit a much smaller optical density across the range of stoichiometries. **b** Magnification of data at pH 3 given in (**a**), with a peak value at a 60:40 mixing ratio. This optical density peak is much lower, but is present across a broader range of stoichiometric ratios. Error bars represent the standard deviation between measurements.

Stock solutions of pMPC and pAA were mixed together over a series of varying stoichiometric ratios. Turbidity measurements, performed at set pH values, are plotted in Fig. 4a, b. At pH = 2, a sharp, clearly defined peak (Fig. 4a) coincides with a pMPC:pAA stoichiometric ratio of 30:70, indicating that in these conditions, an excess of carboxylic acid is needed to interact with a given amount of zwitterion. This means that relatively fewer pAA monomers are available for complexation, as compared to pMPC. Interestingly, a secondary peak at 80:20 occurs at pH 2. At this time, the provenance of this peak is unclear, owing to the difficulty of performing scattering experiments to determine solution structures on such turbid samples. At pH 3 (Fig. 4b, which is a magnification of the results in bottom trace of Fig. 4a), the peak position itself is shifted rightwards, corresponding to a pMPC:pAA stoichiometric ratio of 60:40. This peak is also much broader than that in the pH 2 case, indicating a reduction in the sensitivity of the pH 3 samples to the mixing stoichiometry.

**Fig. 5:**
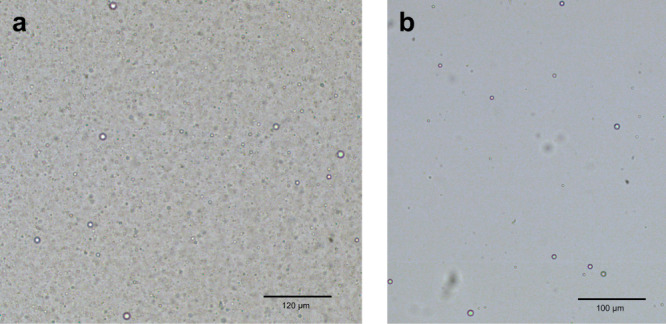
Microscopic appearance of pZCs at pH 2 and 3. **a** Complexes with spherical morphology at pH = 2. Images were taken with a polarized objective lens at 10X magnification. This is a representative image, corresponding to the letter A in Fig. 6, a 30:70 pMPC:pAA stoichiometric ratio. Scale bar corresponds to a length of 120 μm. Note the clear resemblance to traditional polyelectrolyte complexes. **b** Complexes with spherical morphology at pH = 3. Images were taken with the same polarizer objective lens at 10X magnification. This is a representative image, corresponding to the letter B in Fig. 6, a 60:40 pMPC:pAA stoichiometric ratio. Scale bar corresponds to a length of 100 μm. Complexation in these conditions is much less abundant than in those seen in (**a**).

**Fig. 6:**
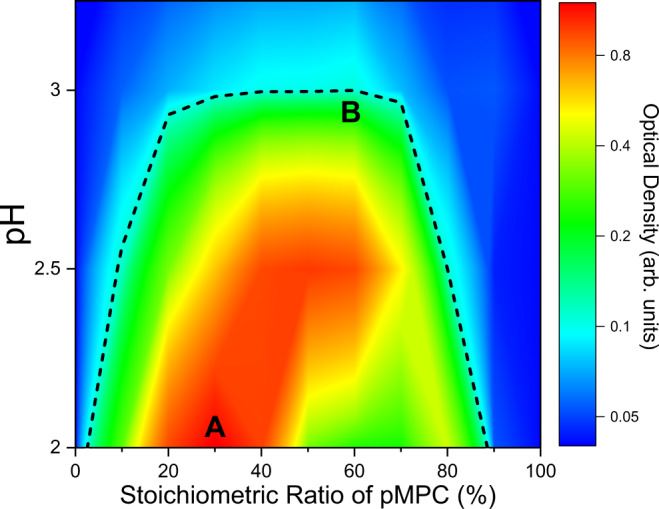
Compiled phase diagram comparing mixing stoichiometry (% pMPC of the total mixture, abscissa) with solution pH (ordinate), taken from experiments like those of Figs. 4 and 5. Color axis indicates the value of the turbidity. Minimal to no complexation occurs above and outside the black dotted line as determined by optical microscopy. Red regions represent the greatest levels of complexation, and dark blue represents no detectable complexation. Results depicted in this diagram are consistent with findings from microscopy. An optical micrograph image of the sample marked by the letter A is seen in Fig. 5a, and the sample corresponding to the letter B is seen in Fig. 5b.

This finding points to a decrease in the availability of pMPC for complexation at pH 3, since at these conditions, fewer pAA chains need to interact with pMPC to produce peak turbidity. Additionally, the relative heights of the turbidity diagrams indicates that more complexation occurs at pH 2 than at pH 3. Quantitatively, it is helpful to note that the relative difference in height between the peak turbidity at pH 2 (30:70 ratio) and at pH 3 (60:40) is approximately one order of magnitude. The arguments in the next section account for this difference, but a cursory comparison of the two micrographs in Fig. 5a, b shows that the number of droplets in the pH 2 case far exceeds the number that is seen in the pH 3 samples, in agreement with the results from turbidimetry. Furthermore, noting that there is a pH difference of one unit, indicating a difference in the proton concentration of an order of magnitude, this result fits neatly with the idea that the observed phenomenon is a consequence of the ionization equilibria of the constituent chains. For pH values at and exceeding 4, complexation is completely diminished, and indeed, micrographs at these conditions indicate the total absence of complexation. These results are compiled and summarized in the phase diagram depicted in Fig. 6.

A question that may arise from the results thus far is whether the droplets in Fig. 5 contain both pMPC and pAA, or if one component in the solution undergoes self-complexation. To address this point, we took care to ensure that both polymers participate in forming droplets. Firstly, a solution of pure pMPC was subjected to increasing concentrations of HCl. As the pH of the solution decreased, there was no change in the turbidity. The turbidity of pAA alone was also insensitive to changes in the pH. No pH-induced self-complexation takes place in either solution component. Next, we wanted to confirm the presence of both pMPC and pAA inside the polymer-rich phase, which is corollary to the absence of self-complexation. Upon formulation of a representative solution (we used the peak 30:70 pMPC:pAA mixing ratio), we spun the polymer-rich phase in a centrifuge, and removed the polymer-poor supernatant. After lyophilizing the solvent from the remaining sample, NMR measurements probed for the presence of each polymer. As expected, evidence from both the pMPC and pAA was seen in the resulting spectra, which proves that both chains are participants in the complexation process described herein. These results are summarized in Supplementary Fig. 5.

Next, to demonstrate definitively that the nature of polyzwitterionic complexation is sensitive to changes in pH and stoichiometry (as is the case in canonical polyelectrolyte complex coacervate systems) optical micrographs were systematically recorded over a range of stoichiometric ratios and pH values. There is significant agreement between the phase diagram plotted in Fig. 6, and the qualitative appearance of the samples under microscopic examination. When present, complexation is characterized by the presence of mobile, spherical droplets of a highly disperse length scale encompassing the sub-micron to tens-of-microns range.

Figure 5a, b are two representative optical micrographs of polyzwitterionic complexes taken from the samples with the peak turbidity values at pH 2 and 3. The labels A and B in Fig. 6 correspond to these two samples, namely, the 30:70 sample for pH 2, and the 60:40 ratio for pH 3, respectively. Our findings show the presence of complexation in almost all samples at pH 2, whereas at pH 3, only some of the samples exhibit spherical droplets, near the peak pH value. These findings bolster the turbidimetry results: the ratios at which complexation is observed are also those with the highest turbidity values. Furthermore, when comparing pH 3 images to pH 2 images, one sees that there are fewer complexes present in the pH 3 samples, regardless of the stoichiometric ratio. This finding agrees with the correspondingly lower maximum turbidity at pH 3 than at pH 2, since the total scattered light intensity is responsive both to size and number of scatterers present. Though comparisons of the number of droplets can be made relatively easily between the two images, acquiring robust measurements of droplet size is nontrivial for numerous reasons. Most importantly, although droplets form immediately upon mixing, they also coalesce over time, and the kinetics of this process is influenced by many factors. Standard size characterization techniques must thus be modified significantly to measure quantities such as the R_*g*_ and R_*h*_ of chains within these droplets, etc., and are hence relegated to a future work. That said, preliminary measurements of droplets using ImageJ software yields estimates of the droplet sizes, with the pH 2 sample containing populations of droplets across a very wide distribution of length scales centered around 2.1 microns, and the pH 3 containing far fewer droplets over a narrower length scale centered around 1.65 microns. Lastly, at pH 4, no demonstrable evidence of complexation is seen at any of the stoichiometric ratios. Hence, we see multiple lines of physical evidence that prove that the phase behavior of complexes made of pMPC and pAA are responsive to their chemical milieu, as would be expected from traditional polyelectrolyte coacervates.

**Fig. 7:**
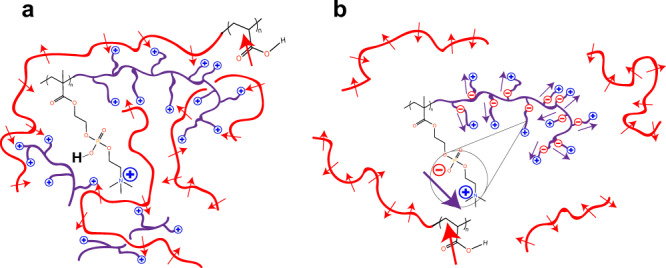
Mechanism for pH-dependent phase behavior of pMPC-pAA complexes. Dipolar pAA chains are depicted in red, and the polyampholyte pMPC is drawn in purple. The corresponding charges of each chain are drawn as red negative symbols and blue positive symbols. In the case of pMPC, charges are tethered to monomers further from the chain backbones, and are therefore drawn as appendages, with the negative charge proximal to the backbone, and the positive charge distal to it. pAA chains can be more closely approximated simply as a line of dipoles due to their smaller monomer size, and much larger chain length. **a** pH < 4: The ionization equilibrium favors the protonation of the pMPC phosphate groups, which confers a formal charge of +1 for each pMPC monomer. In this scenario, the exposed positive charges on pMPC can interact with dipoles along the pAA chains, promoting complexation, and accompanied by counterion release. **b** pH > 4: the phosphoryl groups of the pMPC are mostly deprotonated, effectively neutralizing the positive charge on the choline group. In this state the net charge of each pMPC monomer is zero, and thus there is no favorable interaction promoting association with pAA, owing to the relative weakness of the dipole-dipole interaction compared to the charge-dipole interaction.

These findings, namely, the non-monotonic dependence of turbidity on the stoichiometric ratio between the two polymers, and the presence of a rich array of spherical droplets of many sizes in numerous samples, proves that at sufficiently low pH values, pMPC can combine with pAA to form a new class of material, analogous to those of the complex coacervate family. Furthermore, their phase behavior is orthogonal to that of traditional polyelectrolyte complexes. In other words, at low pH, the pZCs are stable, but in less acidic conditions, the polymers can no longer interact, leading to disassembly. This behavior contrasts with that of traditional polyelectrolyte coacervates, in which complexation only occurs at intermediate pH, and disassembly at very low and very high pH values (Fig. 1). The mechanism of this orthogonal phase behavior, depicted in Fig. 7, and its origin in the zwitterionic component, is described in the following section.

### Mechanistic underpinnings of orthogonal phase behavior of polyzwitterionic coacervates

To understand the phase behavior of pMPC-pAA complexes, one needs to begin by considering the proton association-dissociation equilibrium of each polymer. This equilibrium dictates the total available charge on each type of polymer chain, which in turn controls the availability of each polymer to undergo complexation with a prospective partner.

In the case of pAA, each chain can either be negatively charged in the deprotonated state, or neutral in the protonated state. The number of chains in either state is dictated by the p*K*_*a*_ of the acid group, which was measured to be 6.2. For pMPC, this equilibrium is complicated by the fact that there are two charged groups in each monomer. The choline moiety is positively charged, and since it is in a fully quaternized state, it does not undergo typical acid-base reactions. Hence, the choline moiety carries a permanent positive charge. On the other hand, the phosphoryl group can accept or donate a proton depending on the pH of the solution.

When the pMPC phosphoryl group is deprotonated, its negative charge neutralizes the positively charged choline group. Monomers in this state contain an effective charge of zero. However, when the phosphoryl group is protonated, its negative charge is obscured from the choline group. As a result, monomers in this condition have a formal charge of +1. In solutions that promote the protonation of pMPC, the positively charged choline group can interact with any available pAA chains. The specific pH range in which pMPC is ionized is thus dictated by the p*K*_*a*_ of its phosphoryl group, which was measured to be 2.3.

Taken together, it is possible to explain the turbidimetry and microscopy results. Using the Henderson-Hasselbalch equation, we calculate that at a pH of 2, the proportion of pMPC that is positively charged is close to 80%. The proportion of positively charged monomers of pMPC decreases as the pH increases, to 28% at pH 2.5, 3.8% at pH 3, and so on. From this trend, it is apparent why lowering the pH leads to an increase in complexation. As the pH decreases, more pMPC chains are available in their positively charged state for interaction with the pAA in the solution.

With respect to the data, the higher scattered light intensity and larger number of visible complexes at pH 2 than at pH 3 are accounted for by the mechanism proposed herein. By looking at the proportion of the charged pMPC groups at pH 2 and 3, and factoring in the stoichiometry of the peak samples, one can conclude that there should be a difference of one order of magnitude between the peak turbidities at the two pH values. At pH 2, taking 30% (the peak stoichiometric ratio of pMPC) of the 80% of monomers that are positively charged, one sees that 24% of the sample is composed of charged pMPC units. At pH 3, the peak stoichiometric ratio is at 60%, and taking that proportion of the 3.8% of the pMPC in a charged state, one sees that 2.3% of the overall sample is composed of charged pMPC monomers. This order-of-magnitude difference (24% vs. 2.3%) correlates closely with the difference between peak turbidity values of the pH 2 and pH 3 experiments. Furthermore, the rightward shift in the stoichiometry peak, from 30:70 at pH 2 to 60:40 at pH 3, can be accounted for by the decreased charging of the pMPC at higher pH values. Fewer charged groups on pMPC translates to fewer pAA chains required for interaction. These findings are outlined conceptually in Fig. 7.

Reflecting upon the theory proposed in the Introduction of this work, the specific nature of the interaction between pMPC and pAA can be found using the energetics of interaction between charges and dipoles. As discussed earlier, when MPC is at a low pH, it behaves as a cation, and the interaction between it and acrylic acid is modeled as a charge-dipole interaction. When the ambient pH is raised to 4 and above, the MPC becomes dipolar, while the acrylate group remains dipolar as well. Hence, in this higher pH condition, the energetics can be modeled using dipole-dipole interactions. As shown in Fig. 2e, f, the latter interactions are almost an order of magnitude weaker. Consequently, stable complexes cannot form unless the pH is low enough.

As noted above, it appears that the complexation behavior of the system depends most strongly on the ionization of the pMPC component. Although the above explanation is self-consistent, another subtlety must be added to appreciate the data more fully. Recall that the process of polyelectrolyte complexation is entropically driven by the release of counterions into bulk solution. The explanation for the mechanism of complexation in this section hitherto focused on electrostatic principles that chiefly pertain to enthalpic parameters. These considerations are important for understanding how the chains look as they associate, and what conditions they must adopt in order to participate in complexation in the first place. To understand why they associate, we can now use the above prerequisite information to describe the key entropic contribution to our phase separation process.

Initially, the counter-anion associated with the positively charged amine group on the zwitterion is tethered, preventing these small ions from exploring their full translational entropy in solution. However, as two chains approach one another, the complementary monomer units can interact with one another, instead of their respective counterions, leaving the previously associated counterions free to explore the bulk solution. The complexation of each pair of monomer units results in an increase in the entropy of the small ions per association. This yields a net increase in the entropy of the system. This reaction, so to speak, can roughly be summarized as follows:

$$	pMPC-{{{{{\rm{POH}}}}}}-{{{{{{\rm{N}}}}}}}^{+}{{{{{{\rm{X}}}}}}}^{-}+{{{{{\rm{HOOC}}}}}}-pAA\to pMPC-{{{{{\rm{POH}}}}}}\\ 	\quad-{{{{{{\rm{N}}}}}}}^{+}{{{{{\rm{HOOC}}}}}}-pAA+{{{{{{\rm{X}}}}}}}^{-}$$

As this reaction proceeds in the forward direction, *X*^−^ ions are released into the solution.

This finding leads us to a crucial consequence of the mechanism proposed herein. As long as the pMPC can maintain a net positive charge, complexation will occur. However, if pMPC is a chain of pure dipoles with net zero charge, not only is complexation disfavored, it is a non-starter (N/A). Writing the reaction as before, we see

$$pMPC-{{{{{{\rm{PO}}}}}}}^{-}-{{{{{{\rm{N}}}}}}}^{+}+{{{{{{\rm{X}}}}}}}^{-}+{{{{{\rm{HOOC}}}}}}-pAA\to {{{{{\rm{N}}}}}}/{{{{{\rm{A}}}}}}$$

Clearly, the prospects for phase separation are absent when pMPC is in a neutral dipolar configuration. There is nothing to promote forward progress of the reaction above. The pMPC is uncharged, and thus has no negative counterion to release if brought to close proximity to a pAA monomer.

The augmentation of electrostatic attraction by entropy-dependent behavior of the counterions and chains depicted above is commonly found in coacervates. Indeed, an ever-growing body of literature supports the counterion release hypothesis as the driving force for the complexation between polyelectrolytes. Furthermore, the extension of counterion release as a driving force for polyzwitterionic complex formation is not only consistent with existing bodies of evidence, but also indicates that there is a certain generalizable universality of this mechanism in driving the phase behavior of all types of charged macromolecules.

### Suitability for physiological temperature and pH criteria

**Fig. 8:**
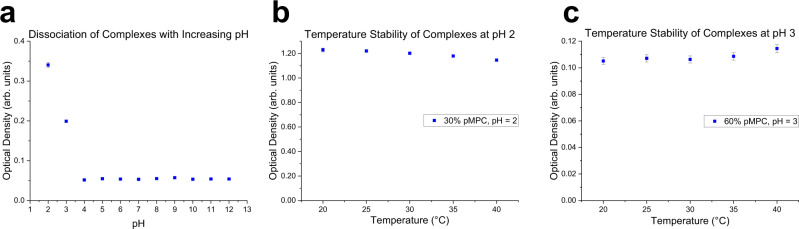
Effects of pH and temperature on pZC assembly. **a** Scattered light intensity as a function of solution pH of 50:50 stoichiometric ratio pMPC:pAA. **b** At pH 2, in samples with a 30:70 stoichiometric ratio (corresponding to the peak), heating over a temperature ranging from 20–40 ^∘^C causes negligible changes in coacervate turbidity, demonstrating stability of pMPC:pAA complexes with respect to temperature. **c** Peak samples at pH 3 (60:40 pMPC:pAA stoichiometric ratio) exhibit a similar resistance against thermal fluctuations over the same 20–40 ^∘^C range. Error bars represent the standard deviation between measurements.

For this system to be pertinent in a biomedical context, we tested it against three criteria, chosen based on the physiology of the gastrointestinal tract. Firstly, the system must remain intact at low pH. Second, the system must be unstable at intermediate and high pH values. The latter criterion accounts for the environment of the duodenum, in which Brunner’s glands facilitate the secretion of basic fluids that neutralize the acidity of the incoming chyme from the stomach (Fig. 1b). Lastly, the complexes must remain intact over a wide temperature range, including that of the GI tract. Figure 8 demonstrates that the pMPC-pAA complexes satisfy these three fundamental requirements. In Fig. 8a, we observe high scattered light intensity readings at low pH, indicating stability in the gastric range. We also see a precipitous decline in the scattered light intensity as the pH reaches 4. Thus, complexation is ideally poised to occur at the pH of the stomach, followed by dissociation at the higher pH of the duodenal space.

To satisfy the third criterion, pZC temperature stability was examined by choosing the highest turbidity peak values from the stoichiometry studies in Fig. 4, and by varying the temperature of each sample from 20 ^∘^C up to 40 ^∘^C in 5 ^∘^C increments. The results in Fig. 8b, c demonstrate that the polyzwitterionic complexes satisfy the third criterion—the temperature does not exert a strong effect on the levels of complexation between pMPC and pAA. These complexes largely resist fluctuations in their thermal environment, a necessary condition when considering the possibility that a material used in the gastrointestinal context may undergo large temperature changes as it travels into and through the body.

### Physical simulation and cargo encapsulation using polyzwitterionic complexes

**Fig. 9:**
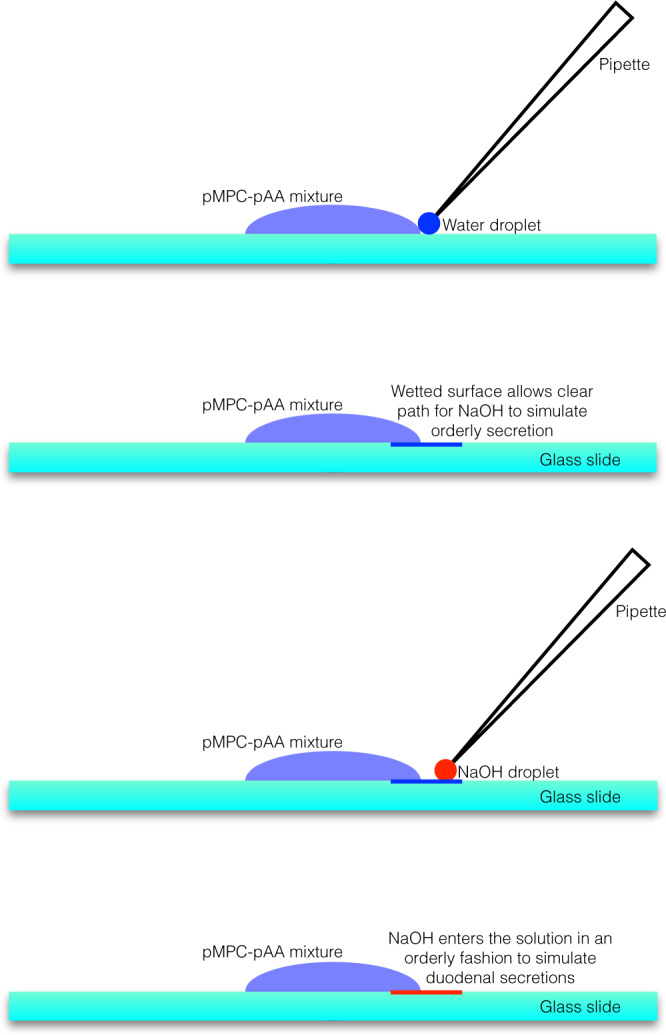
Physical simulation to expose pZCs to physiological conditions relevant to the duodenum. Addition of water to the edge of the droplet (top, blue dot) allows wetting of glass surface along a line near the circumference of the pZC mixture (depicted as a blue line). Next, a small volume of NaOH solution (red dot) is micropipetted at the edge of the wet line created previously. This system in turn promotes the orderly movement of NaOH into the polymer mixture unidirectionally and gradually.

**Fig. 10:**
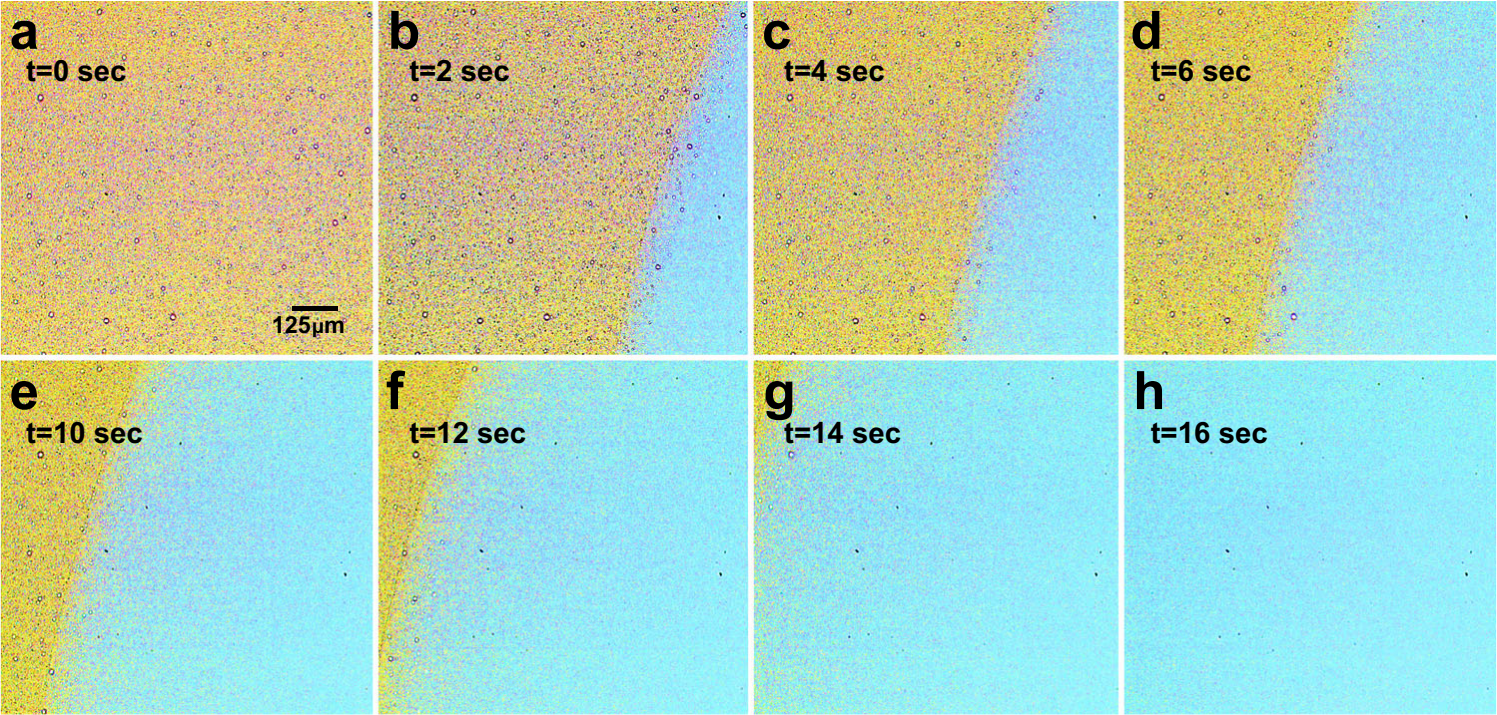
Compilation of video microscopy image frames of NaOH dissolving the pMPC-pAA complexes. A very small volume of NaOH solution was dropped beside a drop of the polymer mixture (in yellow), and the resulting solvent front (cyan) was recorded using a polarized objective with 10X magnification. Frames at 2 s intervals were captured using the Phantom PCC imaging software, and presented in order from panel (**a**–**h**). The scale bar in panel (**a**) corresponds to a length of 125 μm, and is consistent throughout the frames. Images have been enhanced and recoloured for the sake of clarity.

In order to test the non-equilibrium responsiveness of pMPC-pAA complexes to a variable pH, a setup was constructed (Fig. 9, detailed in the Methods section) that allowed exposure of the polymers to a basic solution in a controllable fashion. This method is intended to simulate the effects of the secretion of basic buffer by glands in the duodenum. As the basic solution is added to a large droplet containing the pMPC-pAA mixture, it flows from one side of the droplet to the other in a progressive fashion. Figure 10 summarizes videographic evidence of the pH-induced phase instability of the pZC solution. As NaOH travels from right to left, it dissolves the polymer droplets in its wake. The individual droplets appear to temporarily expand and rupture, until they are fully de-complexed. Control experiments that replace NaOH solution with pure water do not show any such response of the complexes to the added water, which proves that this effect is not due simply to dilution, but to the increase in the ambient pH. Interestingly, we found that the pH-dependent dissociation is reversible: after the addition of NaOH to a solution of pZCs, the complexes dissolve. If HCl is then titrated into the same solution, the pZC droplets return.

It is worth comparing the experiment of Fig. 10 to that of Fig. 6. The two experiments differ when considering the distinction between equilibrium and non-equilibrium behavior with respect to pH. Whereas the measurements in Fig. 6 are made on eleven separate samples prepared over a range of discrete and static pH values, Fig. 10 depicts the dissolution of a single sample whose pH was initially set at 2, and then continuously increased by exposure to a basic solution. In other words, Fig. 6 provides equilibrium information about the pMPC-pAA complexes, while, Fig. 10 demonstrates that the spherical polyzwitterionic complexes exhibit non-equilibrium (with respect to pH) and responsive properties upon exposure to varying physiologically relevant conditions. The activity showcased in Fig. 10—dissolution of the complexes upon exposure to basic media—highlights the responsive nature of polyzwitterionic complexes, and points to their potential usefulness as platforms for gastrointestinal drug delivery.

Having established the above set of activities, we looked to encapsulate cargo into the pZC droplets. To do this, we used a fluorescently labeled protein, BSA (Bovine Serum Albumin). We added the protein in between the additions of pMPC and pAA, so that droplet formation could occur in the presence of BSA. Solutions prepared in this manner appeared turbid as before, and carried a slight color, corresponding to the fluorophore. We spun the ternary solutions in the centrifuge to assess whether the protein co-localizes with the polymer. Optical density measurements of the supernatant (at 495 nm, which is the absorption wavelength of the fluorophore) demonstrated a complete absence of protein in the polymer-poor phase in low pH conditions. By comparison, when the labeled BSA alone was centrifuged in the same spin conditions, absorbance spectra of the conjugated protein were still apparent in the solution, indicating that the protein did not simply sink to the bottom of the tubes during spinning along with the polymers. This experiment was repeated as a function of pH, to assess whether the zwitterionic carriers can release their cargo. Absorbance measurements of the supernatant clearly demonstrate the increased upper-phase BSA concentration as the pH increases from 2 to 5. Hence, there is a pH-dependent release of cargo, as is expected from a carrier that dissolves as a function of pH. Finally, fluorescence micrographs directly demonstrate the localization of the protein into the pZC droplets. These results are summarized in Supplementary Figs. 1–5.

## Discussion

We have shown the existence of complexation between pMPC and pAA. These complexes demonstrate many of the same properties as those seen in polyelectrolyte coacervates, even though one of the components is a polyzwitterion, rather than a traditional polyelectrolyte. We have optically confirmed the presence of the characteristic spherical assemblies seen in coacervate solutions, and demonstrated their sensitivity to their chemical environment and mixing stoichiometry. Furthermore, we have exhibited the orthogonal phase behavior that results from asymmetric charging of the two constituent polymers, and proposed a mechanism that explains this behavior. Lastly, we harnessed these properties to demonstrate the potential applicability of polyzwitterionic coacervates as platform technologies for the pH-triggered release of cargo. This work inspires numerous follow-up questions of both a fundamental and applied nature.

An implication of the mechanism depicted in the Section titled Mechanistic underpinnings of orthogonal phase behavior of polyzwitterionic coacervates of the Results is that the specific acid-base chemistry of the monomers, particularly the zwitterionic monomer, dictates the exact range of acid-stability and base-lability of the polymeric complexes. Indeed, different polyzwitterions may yield phase behavior that is shifted around different pH values relative to that of the pMPC-pAA system at hand. Furthermore, it may be possible to design systems that exhibit inverse phase behavior relative to what is seen here, namely, base stability and acid lability. Future work may demonstrate the feasibility of engineering such phenomena using complementary synthetic approaches.

Fundamentally, aspects of the phase behavior presented here have implications in complexation within other polyzwitterionic materials. Designing analogous synthetic polyzwitterions to tailor specific pH-responsive criteria could be one avenue of fruitful research. The chemistry can be adjusted to suit stability or instability requirements at particular pH values. Furthermore, switching the two charged groups on the zwitterionic monomer, such that the permanently charged functional group is negative, and the variably charged functional group is positive, should lead to inverse phase behavior relative to that described in this work, if such a polyzwitterion were paired with a traditional polycation. Of course, the findings in this work pertain also to naturally-occurring zwitterionic materials, namely proteins. Designing conjugates or adjuvants that interact with proteins to promote self-assembling structures must take into account the phenomena outlined in this work.

In more applied contexts, the type of pH-dependent phenomenology described herein can be engineered into existing modalities of drug transport and delivery. Matters regarding the specific formulation of a hybrid therapy remain open, as do questions about the types of therapies that are best suited for encapsulation by polyzwitterionic assemblies. Furthermore, in the relatively chaotic environment of the GI tract, one must take the influence of additional factors, such as other complexing agents, and physiological parameters beyond the pH and temperature (such as ionic strength), into account. The list of open questions is extensive, especially considering the specifics of a given pharmaceutical class, a set of drugs within that class, and targets for those drugs. We hope that these ideas lay the groundwork to tackle the imminent problem in pharmacology of gastrointestinal drug delivery.

## Methods

### Materials and synthesis

Monomeric MPC was purchased from Sigma Aldrich (MilliporeSigma, Burlington, Massachusetts, USA) (Fig. 3a). 1 g of 2-methacryloyloxyethyl phosphorylcholine (MPC) was dissolved in 3 mL of deionized water. To this was added 19 mg 4-cyano-4-(phenylcarbonothioylthio)pentanoic acid dissolved in 0.4 mL methanol and 0.1 mL of a 20 mg/mL stock solution of 4,4’-azobis(4-cyanovaleric acid) (ACVA) in methanol. The reaction mixture was degassed by purging with N_2_(g) under ice bath cooling for 30 min. The solution was heated at 70 ^∘^C for 6 h, then quenched by rapidly cooling with liquid nitrogen and opening the reaction vessel to air. The crude product was purified via dialysis against water and subsequently freeze-dried to afford the pure product as a light pink solid in 86% yield with a molecular weight (M_*n*_) of 22,000 g/mol. Molecular weight analysis (M_*n*_, M_*w,*_ and D) was carried out via gel permeation chromatography (GPC) against PMMA calibration standards, using an Agilent 1200 series system equipped with a degasser, RI-detector, PFG guard column (8 × 50 mm) and PFG analytical linear M columns (8 × 300 mm, particle size 7 mm) from Polymer Standards Service, and an isocratic pump. The eluent was 2,2,2-trifluoroethanol (TFE) containing 0.02 M sodium trifluoroacetate, with a flow rate of 1 mL/min at 40 ^∘^C. ^1^H-NMR studies of the synthesized pMPC (500 MHz, D_2_O, *δ*) reveal the following shifts: 8.05 - 7.50 (CTA), 4.40 - 4.00 ppm (6H), 3.71 ppm (2H), 3.27 ppm (9H), 2.20 - 0.80 (5H+CTA). Spectrum can be found in Supplementary Fig. 6.

pAA (Fig. 3b) was purchased (CAS# 00627-250, Polysciences, Inc., Warrington, Pennsylvania, USA) as a 25 wt.% solution and diluted in Milli-Q water to make a 1 wt.% stock solution. It has a molecular weight of 50,000 g/mol, and was used without further modification or purification after dilution. HCl (*aq*) and NaOH (*aq*) were used to adjust pH values of stock solutions.

### Preparation of pAA and pMPC samples

pAA and pMPC stock solutions were prepared by dissolution of dry polymer powder (pMPC) or concentrated polymer solution (pAA) into Milli-Q water (18.2 MOhm ⋅ cm resistivity at 25 ^∘^C, MilliporeSigma, Burlington, Massachusetts, USA). Stock solutions were set to a concentration of 1 weight percent. The stock solution pH was adjusted to a range spanning from 2 to 12 in increments of 1 pH unit using small volumes of concentrated HCl or NaOH. Stock solutions of the pAA and pMPC were mixed at specified stoichiometric ratios in increments of 10% by micropipetting them into microcentrifuge tubes (Thermo Fisher Scientific, Waltham, Massachusetts, USA) containing a specific volume of Milli-Q water to reach a final total polymer concentration of 0.75 wt.%. pMPC was added before the pAA, and this mixing order was kept consistent throughout the measurements. Samples were vortexed at high speed for 10 s between each step to ensure thorough and consistent mixing.

### Optical density

The optical density, or turbidity, or total scattered light intensity of each sample was measured by a Tecan Infinite 200 Pro instrument (Tecan Group Ltd, Männendorf, Switzerland). Optical density can be determined from the equation T $={\log }_{10}\frac{{I}_{\circ }}{I}$, where *I*_∘_ is intensity of the light unattenuated by the sample, and I is the intensity of the light that is transmitted through the sample. Thus, a reading of T = 0.35 indicates that the sample causes an attenuation of the light intensity by a factor of 10^0.35^, or 2.24-fold. In other words, only 44.7% (100 ÷ 2.24) of the *I*_∘_ light that is incident upon the sample reaches the detector. Measurements were performed by placing samples within clear, flat-bottomed polystyrene 96-well plates (Corning Inc., Corning, New York, USA), and scanning with 550 nm light, since samples do not absorb light at this wavelength. Each sample was shaken within the plate reader before measurement as a final step to ensure uniform mixing among batches. Turbidity measurements were performed in a temperature range spanning from 20 ^∘^C to 40 ^∘^C in 5 ^∘^C increments.

### Optical microscopy

Optical micrographs of small aliquots of each sample were recorded on a Leica DM 2700P instrument with a single polarizer (Leica Camera AG, Wetzlar, Germany). Samples were inspected on Fisherbrand glass slides (Thermo Fisher Scientific, Waltham, Massachusetts, USA) under 10× magnification. Phantom PCC 3.5 software (AMETEK Inc. Vision Research, Wayne, New Jersey, USA) was used to compile and visualize still image data, and video micrographs were compiled and processed using the Phantom Video Player software, from the same provider.

### Synthetic mimic of dissociation of complexes on glass and video microscopy

To produce conditions by which a controlled and unidirectional application of a basic medium to a solution of polyzwitterion-polyanion complexes could occur, a setup was constructed to visualize the complex dissociation process. Using a micropipette, a relatively large droplet (300 μL) containing the pZCs was placed at the center of a glass slide and allowed to relax for several minutes. Next, a much smaller volume of Milli-Q water (15 μL) was added to the edge of the droplet, such that the two fluids could touch and mix. The pipette tip was carefully positioned at the intersection of the droplet with the glass slide, and a sliding motion was initiated as the plunger was depressed, in order to produce a thin streak of water on the glass. This ensures that the glass slide is wetted at a specific location, allowing for the subsequent addition of another aqueous solution without surface tension preventing even mixing from occurring.

Next, a similarly small (15 μL) droplet of concentrated NaOH solution was added at the apex of the streak left by the pipette tip when the water was previously deposited onto the glass surface. After addition of the NaOH, it spontaneously mixed with the polyzwitterion-polyanion solution exclusively via this channel in an orderly, unidirectional fashion, since the surface energy mismatch between the glass slide and the concentrated aqueous NaOH was large enough to prevent further wetting of the glass. The progression of the basic medium through the droplet, and the subsequent dissociation of the complexes at the solution front, was monitored using video microscopy by carefully positioning the glass slide underneath the polarized objective such that the velocity vector of the dissociation front intersected the light path of the viewing area.

### Titration of pAA and pMPC

400 μL of each salt-free polymer solution was prepared by diluting from stock to a concentration of 0.75 wt.% and added to a 2 mL glass vial with a small Teflon stir bar. A pH probe was inserted into the solution, with the tip of the bulb positioned above the bottom of the vial to provide a sufficient amount of space for the stir bar to adequately mix the solution. The titrant was manually added directly into the polymer solution in 1 μl increments using a micropipette, and a sufficient amount of time (1 min per addition of titrant) was allowed to elapse for the solution to equilibrate before the pH value was recorded.

### Complexation of protein cargo within pZC droplets

Bovine serum albumin (BSA) conjugated with a fluorescent dye (Alexa Fluor^*TM*^) was purchased from Invitrogen (Life Technologies Corporation, Eugene, Oregon, USA) in powdered form. The protein was used as purchased, and dissolved in water. After optimization of loading conditions and concentrations, BSA solution was added in between additions of the pMPC and pAA components of the solution to ensure that complex formation takes place in the presence of protein. Cargo uptake studies were performed by making independent optical density measurements at the peak absorbance wavelength of the protein (495 nm) and that corresponding to the turbidity measurements of the complexes (at 550 nm, as outlined previously in the Methods section) simultaneously. Centrifugation was used to separate the solution into polymer-rich and polymer-poor phases, and the preferential segregation of the cargo was tracked by assessing the absence of protein in the upper polymer-poor phase. (Increased concentrations of protein in the polymer-poor phase indicates decreased levels of complexation.) Confirmatory microscopy images were taken using a Nikon CrestV2 confocal fluorescence microscope (Nikon Instruments, Inc., Melville, NY, USA).

## Supplementary information

Supplemental data

## Data Availability

Additional data that support the findings of this study are available from the corresponding author upon request.
