# Supplementary material for: Coacervation in polyzwitterion-polyelectrolyte systems and their potential applications for gastrointestinal drug delivery platforms
Source: Nat Commun. 2022 Apr 26;13:2250. doi: 10.1038/s41467-022-29851-y (PMC9042848; doi:10.1038/s41467-022-29851-y)
Supplement: Supplementary file 1 — Supplemental data [file 41467_2022_29851_MOESM1_ESM.pdf]

Supplemental Information

Coacervation in polyzwitterion-polyelectrolyte systems and their potential applications for gastrointestinal drug delivery platforms

Khatcher O. Margossian, Marcel U. Brown, Todd Emrick, and Murugappan Muthukumar

These experiments demonstrate the encapsulation of model cargo, BSA, into the pZC droplets. First, the absorbance spectrum fluorescently labeled BSA was measured to determine its peak absorption wavelength (495 nm). The fluorophore was selected such that it has an absorption peak sufficiently distant from the wavelength at which turbidity measurements were collected (550 nm). Next, the three-component system was mixed to yield a solution containing both the protein as well as the pZC droplets. Encouragingly, the bulk solution absorbance in the presence of BSA followed the same pH-dependent behavior seen in the absence of BSA. However, this measurement does not sufficiently localize the BSA to the interior or exterior (or both) of the pZC droplets. In order to test if/where the protein segregates within the solution, the samples were spun down in the centrifuge to induce bulk phase separation, into a polymer-rich lower phase and a polymer-poor supernatant. Because accurate measurement of the turbidity of a viscous and low-volume lower phase is experimentally intractable, absorbance measurements were carried out on the upper phase instead. At pH 2, when complexes form, the upper phase turbidity at 550 nm (corresponding to the polymer droplets) and the 495 nm absorbance were at their minimum baseline value, indicating the absence of both the protein and the complexes. This finding indicates that the protein co-localizes with the polyzwitterion complexes at low pH. However, as the pH was increased to 4 and 5, the upper phase absorption at 495 nm reached levels that correspond to the bulk solution absorbance of the protein of ~0.2 a.u., consistent with the value of the peak seen in figure S1. This finding indicates that as the pH is increased, the protein is released, and thus cannot segregate with the zwitterionic complexes. Finally, to test the preferential segregation of BSA with the zwitterionic complexes directly, we performed fluorescence microscopy to observe the co-localization of the proteins and the chains into droplets. A representative image can be seen in Figure S4.

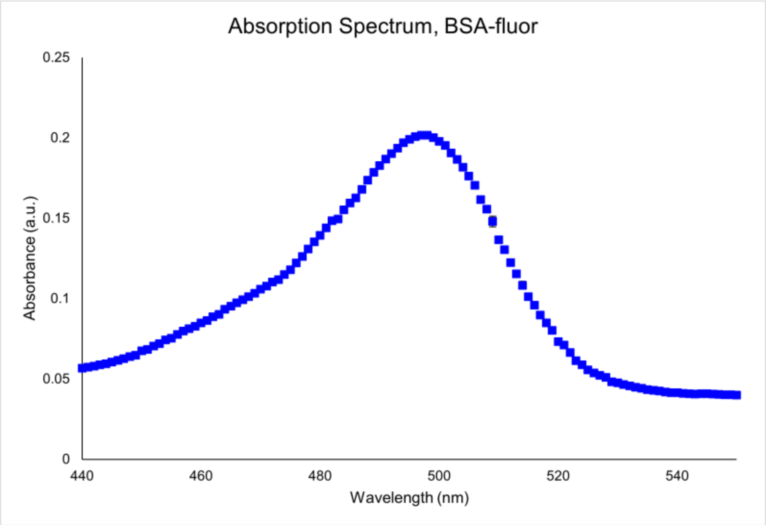

Figure S1: Absorbance spectrum of AlexaFluor-tagged BSA. An absorbance peak at approximately 495 nm indicates the presence of the fluorescently-labeled protein. Note the absence of absorbance at 550 nm, which allows us to track the presence of protein and polymer independently using absorbance spectroscopy.

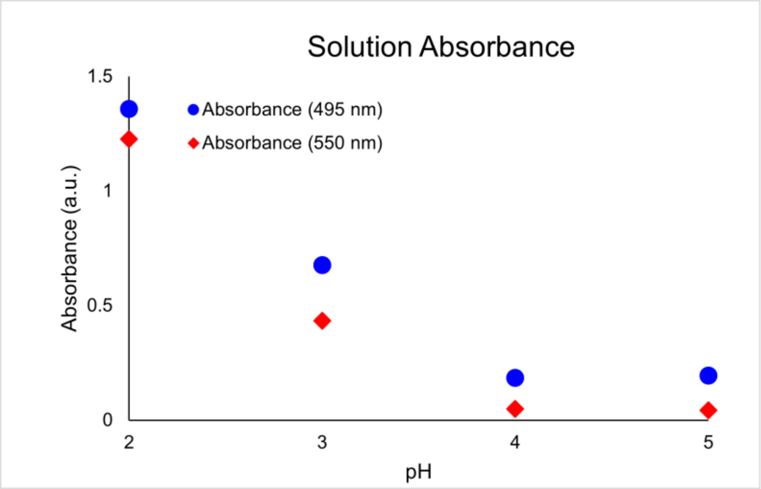

Figure S2: Bulk solutions of BSA-loaded pZCs have the highest absorbance at low pH, consistent with previous results. As pH increases, the pZC assemblies dissolve, and thus their absorbance falls to baseline levels. The presence of pZCs is monitored by the absorbance at 550 nm. Absorbance values at 495 nm are used to track the tagged BSA. As the pH increases and the complexes dissolve, the protein absorbance decreases to its baseline level of approximately 0.2 a.u. This value is much higher than the baseline reading for the polymers at 55o nm, which is typically between 0.04 and 0.05 a.u.

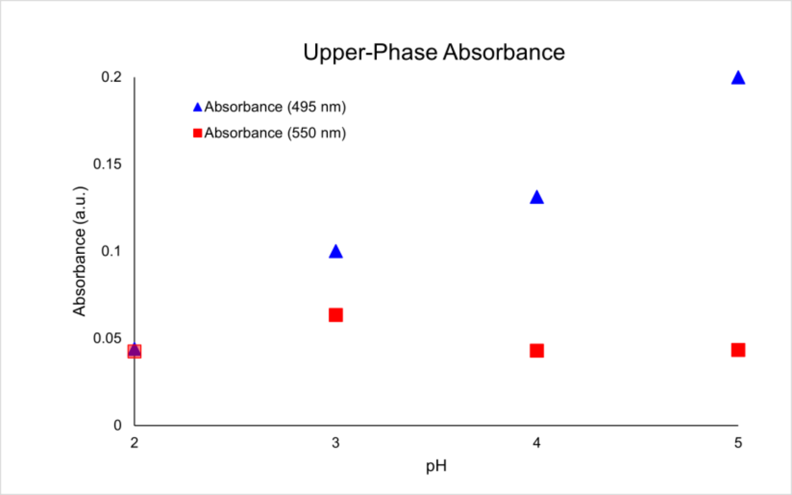

Figure S3: After centrifugation of the BSA-loaded pZCs, the supernatant absorbance was measured as a function of pH. The absorbance at 550 nm (red squares) is close to baseline regardless of the measurement, indicating that the polymer assemblies are not present in the supernatant. As the pH increases, the pZCs disassemble. They do not form complexes at pH 4 or 5, but at pH 2 and 3, the complexes they do form are effectively excluded from the measurement, as indicated by the relatively flat values of the 550 nm absorbance measurements. However, as the pZCs disassemble, the absorbance values at 495 nm (blue triangles) increase steadily, until they reach the 0.2 a.u. value that is expected when they are free in solution. (This is the peak absorbance value seen in the initial absorbance curve.) The sub-0.2 a.u. value before pH 5 indicates that there may be some degree of interaction between the polymer chains and the protein in acidic conditions. Nevertheless, the increasing absorbance as a function of increasing pH indicates that BSA is preferentially segregating with the polymer complexes at low pH, and is released as complexes dissolve at higher pH values. The complete absence of BSA absorbance at pH 2 proves that this cargo interacts closely with its carrier, and that as the carrier is made to dissolve, the protein cargo is released into the bulk.

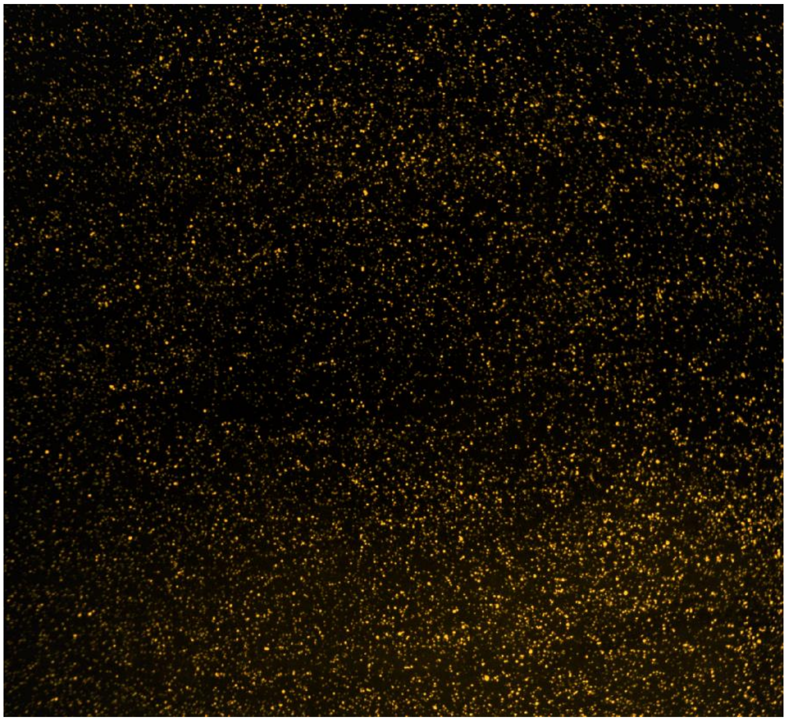

Figure S4: Fluorescence micrograph of AlexaFluor488-conjugated BSA encapsulated within pZC droplets at pH 2. Image clearly demonstrates preferential segregation of the protein into the polymer-rich phase, and absence of the protein in the polymer-poor phase.

NMR study:

Upon formulation of a representative solution (the peak 30:70 pMPC:pAA mixing ratio at pH 2), we spun the polymer-rich phase in a centrifuge, and removed the polymer-poor supernatant. After lyophilizing the solvent from the remaining sample, solid state NMR measurements probed for the presence of each polymer. As expected, evidence from both the pMPC and pAA was seen in the resulting spectra, which proves that both chains are participants in the complexation process. 10% of D_2O added to the solid to enhance the spectral resolution. Cross-polarization (CP) NMR was used to obtain the following spectrum.

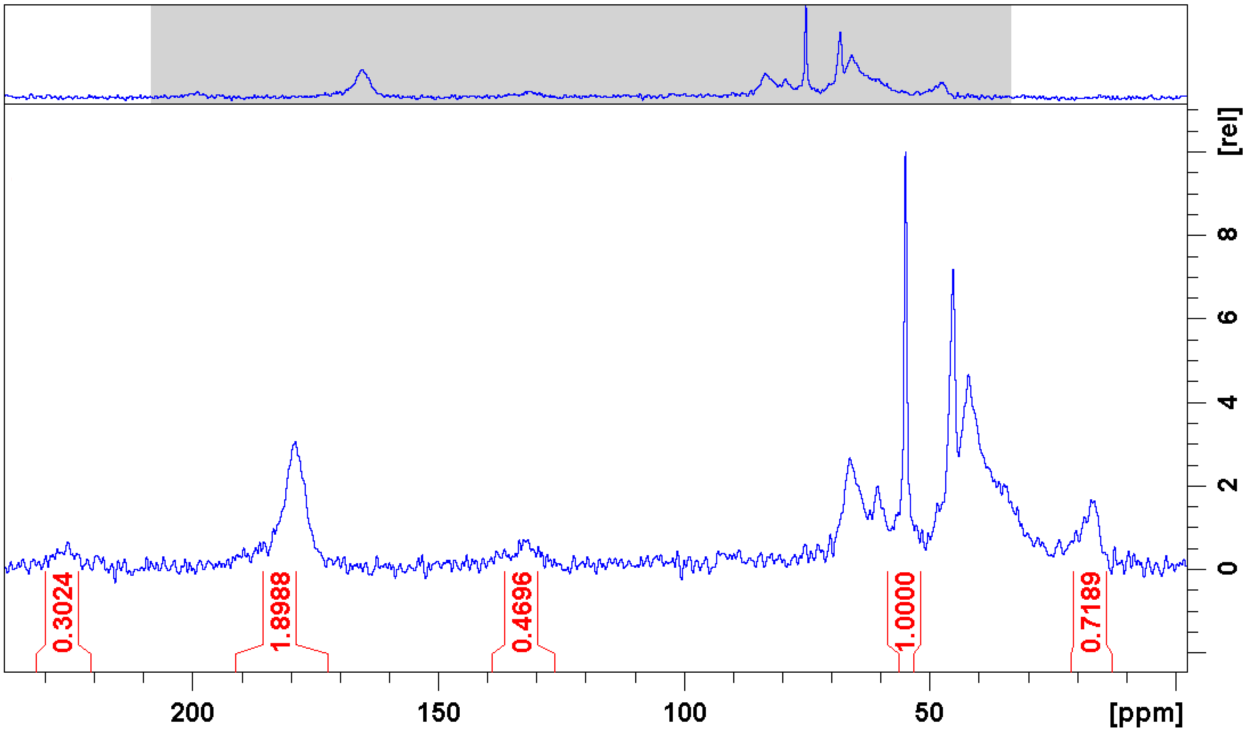

Figure S5: The above figure is the CP spectrum with magic angle spinning speed of 7 kHz and CP time of 1 ms. The 55 ppm peak and the 17 ppm peak are from N-methyl and the backbone methyl in MPC, respectively. The 180 ppm peak is from the carbonyl signal (present in both PAA and MPC), while the small peaks at 130 and 225 ppm are spinning sidebands of the carbonyl. Other peaks can also be appropriately assigned. The backbone methyl instead of the N methyl is used to represent the MPC population as the former has similar molecular dynamics as the PAA carbonyl and thus would be excited to a similar degree in CP experiments. The presence of these peaks indicates that the complexes contain both the pAA and pMPC components.

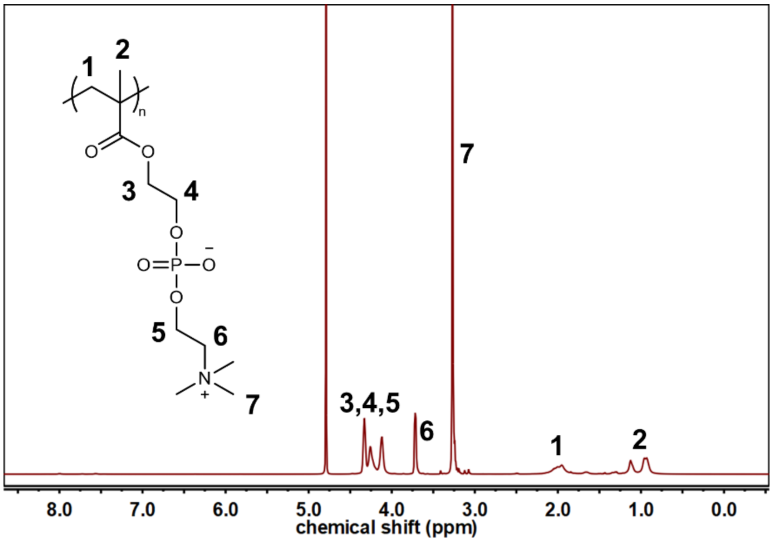

Figure S6: NMR spectrum and associated shifts of pMPC. ^{1}H-NMR (500 MHz, D_2O, δ): 8.05 - 7.50 (CTA), 4.40 - 4.00 ppm (6H), 3.71 ppm (2H), 3.27 ppm (9H), 2.20 - 0.80 (5H+CTA).
